# Should we, can we, halt the rise in prescribing for pain and distress?

**DOI:** 10.3399/bjgp20X712217

**Published:** 2020-08-19

**Authors:** Richard Byng

**Affiliations:** Faculty of Health: Medicine, Dentistry & Human Sciences, University of Plymouth, Plymouth.

## INTRODUCTION

Awareness of prescribed opioid dependence is now reaching the general population along with concerns about levels of antidepressant prescribing and the potential for withdrawal symptoms. Gabapentinoids have become controlled drugs and Public Health England have published their report on prescribed drugs and dependence detailing extensive long-term prescribing.^1^ Family doctors will not have failed to notice both the increasing numbers of patients being prescribed multiple drugs for pain and distress, and the change in tone in consultations as we start to worry about their effects and wonder whether adding more, or another, or just switching drugs is the right action. What is the nature of the problem? What can we do instead?

**Figure 1.**
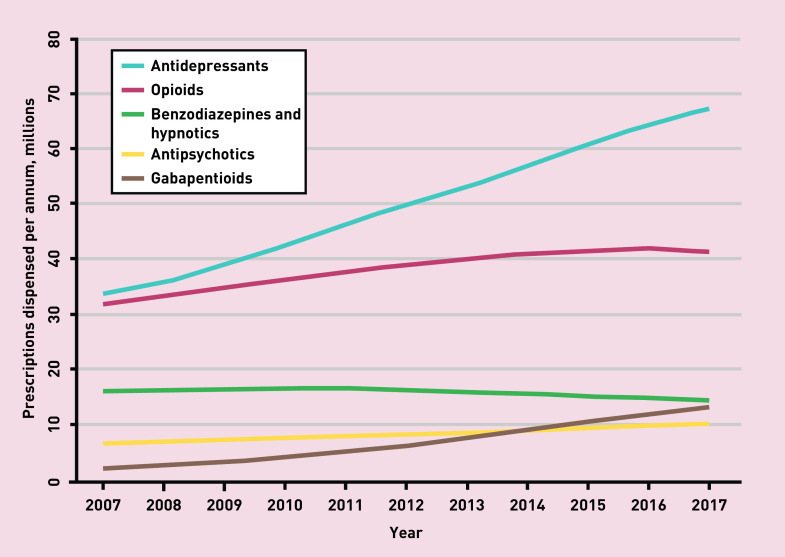
***Prescriptions dispensed per annum by central nervous system drug class, in England, 2007–2017. Developed with NHS Digital data.****^2^*

The increase in gabapentinoids (pregabalin and gabapentin) prescribing in England has been dramatic between 2007 and 2017, from 2.1 to 13.2 million items per year.^2^ Total opioid prescriptions peaked in 2016, though more powerful agents continued to rise.^2^ In the last 10 years, we have seen a considerable rise in the antipsychotic quetiapine (doubling to 3.3 million items/year).^2^ And, the mammoth in the room, a continued decade on decade advancement in total antidepressant prescribing to 68 million items per year^2^ as ‘beyond guideline’ long-term prescriptions increase.^1^ Prescribing rates in some poor post-industrial and coastal areas boast average antidepressant scripting of 2 items per person per year.^3^ Figure 1 depicts the item rises together.

## UNDERSTANDING RISES OVER TIME

To understand these trends on the population over time and the potential solutions, we need to consider: the drug in the body; the patient and the doctor; and the systems and cultures influencing behaviour. These medications act mainly on neuronal tissue with varied short- and longer-term effects. The appeal is for immediate relief of pain-distress, but it is increasingly recognised that rather than solving disease-based chemical imbalances, central nervous system (CNS) facing medications have ‘psychoactive effects’ as a result of changes to neuronal pathways^4^ — short-term benefits or negative effects may then be followed by a homeostatic response to the drug (some form of tolerance), often with a lessening of any benefits or harms. On withdrawal there can be significant symptoms (a second imbalance) due to a reduced ability of synapses to perform their normal function. This broad pattern plays out differently for each drug-body dyad due to pharmacokinetic, pharmacodynamic, and psychological heterogeneity. For antidepressants and antipsychotics the evidence for tolerance is less strong and withdrawal effects appear to be less common.^1^

While there is evidence that short-term prescriptions of analgesics and psychotropics can relieve pain and distress, the evidence for long-term benefit is poor.^5^ Antidepressants have small average positive effects in the short term and with continuation treatment,^6^ but the modes of action are still unclear, and benefits and harms may accrue through sedation or a disconnect from reality.^4^ Evidence from naturalistic studies of selective serotonin reuptake inhibitors (SSRIs) use shows relapse rates of greater than 20% over 2 years and either minimal harm or benefit with discontinuation compared to continuation, depending on measurement method.^7^ There are no long-term randomised studies.^8^

Second-generation antipsychotics, while primarily licensed for psychosis, are being used for mood instability, agitation, insomnia, and anger. Like antidepressants, mechanisms of action are poorly understood. While there is evidence of benefit in the short term for psychosis, the only long-term randomised study shows functional recovery is better.^9^ Pregabalin and gabapentin act through uncertain mechanisms to reduce neuropathic but not back pain,^10^ are potent anxiolytics, often cause dizziness and poor concentration, and have a high risk of misuse in susceptible individuals.^1^ There is less favourable evidence for fibromyalgia, with 30% of those who had initial benefit already relapsing within months despite continuing treatment.^11^ The evidence for the harm of long-term opioid prescribing is substantial and internationally recognised, with guidance suggesting intermittent rather than continuous use in order to reduce tolerance, dependence, and addiction.

The links between mind and body may help explain high rates of co-prescribing and point to solutions. Both the gabapentinoids and opioid analgesics, while primarily prescribed for pain, have effects on emotions (reducing anxiety, while increasing emotional detachment and euphoria). Seeking and providing short-term relief through psychotropic medication has become a norm in primary care. Even if sceptical of the benefits, we can easily end up prescribing multiple classes of CNS medications for the same patient. Distress, pain, adverse childhood events, and social hardship are all correlated. This, and tolerance to individual drugs, help explain why our practices have so many people still in distress and pain despite multiple psychotropic medications.

## REVERSING THE TREND

The evidence for harm is not definitive but we do have a degree of control in our roles as prescribers — as well as a responsibility to consider the whole population, not just the patient before us — and as GPs to act together with pain specialists, physicians, surgeons, and psychiatrists. We can build both on our emerging collective understanding of the problem and on successes from the past. We have achieved reductions in amphetamine and benzodiazepine prescribing in the past,^12^^,^^13^ and more recently addressed over-prescribing of antibiotics and antipsychotics for those with dementia.^14^^,^^15^

It is clear that changing the nature of the consultation, whether at initiation or review, needs to be at the heart of any change of practice. We need, somehow, to gain an understanding of the complex social and emotional factors leading to an individual’s pain and distress, their use of prescribed medications, street acquired substances, and alcohol. Shared decision making has to deal with perhaps the ultimate problem of medical personalisation, that it is in most cases impossible to know whether improvements in outcomes after initiating medication are due to placebo effects, pharmacological effects, or other factors; and whether reductions in effect are due to pharmacological tolerance, loss of placebo effects, or changing circumstances. Perhaps we just have to always presume that improved outcome is less rather than more likely to be due to the drug.

Clear, accessible information to support prescribing and deprescribing will be needed: the evidence of harms and uncertainties alongside relatively small benefits; how the range of psychotropic drugs act via an initial impact on neuronal function, different forms of subsequent adaptation, and a variable intensity of withdrawal; and tapering regimes, perhaps supported for some by publicly available electronic interfaces. Prescribers may want to move towards a ‘selective-use protocol’ based on individualised trial of medication and change depending on immediate and short-term response,^5^ rather than the indiscriminate current ‘offer to all with a diagnosis’ method of practice. Such provision of information and clinical practice will need to be supported, not just by compassionate conversations, but also with organisational and psychosocial approaches. These will need to be evaluated as we proceed. It will also be important to take into account social adversity and work collaboratively with resource poor communities to co-design both consultation approaches that overcome mistrust and community interventions that build on local strengths.^16^

Systems within primary care could include: a more robust approach to repeat medication reviews identifying those needing proactive personalised care; and a multiprofessional approach involving practice and community pharmacists covering the important roles, including person-centred assessment and support. A wider comprehensive and integrated service could include links with community-based interventions through social prescribing, the development of mind-body interventions for tiredness and pain, and the wider availability of psycho-education and psychological therapies addressing emotional lability. In addition, community dialogues about possible harms (both psychological and physical) of medication and the potential benefits of both changes in our previous responses to pain and distress, while politically challenging, may help individuals and communities understand and support such a radical change in approach.

As generalist physicians we need to decide whether to substantially change our practice of providing short-term solace for very real pain and distress. Local initiatives and the measures suggested in the Public Health England report^1^ are just a start. In order to put a halt to what in 20 years might be seen as the age of mass iatrogenic poisoning of the brain, we would need to substantially reduce prescribing and increase deprescribing now, as well as invest in psychosocial interventions rather than wait for more definitive research.
